# Editorial: lncRNAs: application in immunotherapy, radiotherapy, and chemotherapy

**DOI:** 10.3389/fcell.2024.1438773

**Published:** 2024-07-25

**Authors:** Huaifu Cheng, Ulf D. Kahlert, Wenjie Shi

**Affiliations:** ^1^ Department of Anal-colorectal Surgery, Shengli Oilfield Central Hospital, Dongying, China; ^2^ Molecular and Experimental Surgery, University Clinic for General-, Visceral-, Vascular- and Trans-Plantation Surgery, Medical Faculty University Hospital Magdeburg, Otto-von Guericke University, Magdeburg, Germany

**Keywords:** lncRNAs, immunotherapy, radiotherapy, chemotherapy, cancer

Cancer is notoriously challenging due to its high invasiveness and mortality rates, making the development of effective therapeutic approaches critically important (Dermani et al., 2019). The RNA-based treatments for cancer have steadily evolved from theoretical concepts to practical applications (Burnett and Rossi, 2012). Within these therapies, non-coding RNA (ncRNA), which does not encode proteins, demonstrates clinical efficacy against tumors by inhibiting mRNA transcription and interacting with proteins to hinder their function. Long non-coding RNAs (lncRNAs), a subclass of ncRNAs, are defined as RNA molecules that are over 200 nucleotides in length (Mattick et al., 2023). An increasing number of studies have documented that lncRNAs play versatile roles in regulating gene transcription, post-transcriptional modifications, translation, and epigenetic modifications. Dysregulation or dysfunction of lncRNAs is closely associated with various diseases (Fang and Fullwood, 2016). During cancer development, lncRNAs may regulate cell proliferation, apoptosis, migration, invasion, and the maintenance of stemness (Fang and Fullwood, 2016; de Oliveira et al., 2019).

Previous studies have shown lncRNA can regulate immune cell-specific gene expression and mediate immune processes, potentially playing a crucial role in immunotherapy resistance (Wang et al., 2014; Zhou et al., 2019). In addition, some scholars identified a lncRNA, *HOTAIRM1*, is significantly associated with shorter survival in glioblastoma patients, contributing to tumor aggressiveness and radioresistance, making it a potential novel therapeutic target (Ahmadov et al., 2021). Moreover, lncRNA are also recognized for their roles in ovarian cancer biology, influencing responses to various chemotherapeutic agents, including platinum salts, taxanes, and poly (ADP-ribose) polymerase (PARP) inhibitors, and have the potential to serve as biomarkers and therapeutic targets to improve treatment outcomes (Wambecke et al., 2020). Considering the crucial roles of lncRNAs in cancer, therapies based on lncRNAs may offer promising new avenues for cancer treatment. This Research Topic explores novel lncRNA-based tumor therapies, mechanisms of lncRNA regulation of therapeutic sensitivity, and the construction of lncRNA regulatory networks for therapy resistance. Additionally, it includes predictive models for antitumor efficacy, as well as the identification and validation of candidate lncRNAs to forecast prognosis and treatment outcomes, offering a comprehensive overview of the potential of lncRNAs in improving cancer treatment strategies.

Chu et al. identified a module of six cuproptosis-associated lncRNAs, termed CuLncScore from The Cancer Genome Atlas Program (TCGA) sarcoma chort, which effectively predicts sarcoma prognosis and characterizes the tumor immune microenvironment (TME). These findings suggest that cuproptosis lncRNAs play a significant role in sarcoma and could inform personalized therapeutic strategies targeting cuproptosis.

Qiao et al. successfully constructed and validated a competing endogenous RNA (ceRNA) regulatory network for tamoxifen-resistant breast cancer, identifying key genes, microRNAs (miRNAs), and lncRNAs involved in drug resistance from the Gene Expression Omnibus (GEO) database. These findings provide valuable insights and potential therapeutic targets for overcoming tamoxifen resistance in breast cancer treatment.

In another study, Yin et al. identified five chemotherapy-related lncRNAs that predict drug resistance in hepatocellular carcinoma (HCC) with high accuracy, highlighting *CAHM*, coding cell adhesion molecule hemophilic, as a hub lncRNA for chemotherapy resistance. Molecular docking suggested Moschus as a potential candidate drug targeting CAHM, offering new therapeutic avenues for overcoming HCC drug resistance.

Yang et al. also reported a case study highlights the effectiveness of combining pembrolizumab and I-125 seeds brachytherapy (ISB) in treating mesenchymal-epithelial transition (MET) amplification advanced non-small cell lung cancer (NSCLC) resistant to MET inhibitors. Additionally, the identification of lncRNA *AL654754.1* as a key factor in radiotherapy response through pathway analysis underscores its potential role in enhancing precision treatment for lung cancer.

In addition, some scholars have reviewed the roles of lncRNAs in tumors and diseases within this Research Topic, such as Hong et al. reviewed the roles and regulatory activities of lncRNAs in HCC, highlighting their involvement in various stages of HCC progression and their potential as diagnostic and therapeutic targets. Understanding these mechanisms may improve diagnostic sensitivity and specificity and lead to the development of HCC-specific treatments.

Zhang et al. highlighted the role of lncRNAs in gastric cancer (GC) immunotherapy, emphasizing their impact on treatment efficacy and drug resistance. It discussed the mechanisms by which lncRNAs regulate immune-related features and contribute to immunotherapy resistance in GC, offering insights into potential therapeutic strategies.

Ghafouri-Fard et al. summarized the diagnostic and prognostic significance of lncRNA prostate androgen-regulated transcript 1 (PART1) in multiple cancers and non-malignant disorders, including prostate cancer, intervertebral disc degeneration, and Parkinson’s disease, emphasizing the role of PART1 in cell proliferation, migration, and metastasis.

Autophagy can both suppress and promote cancer and increasing evidence shows that dysregulated lncRNA expression could disrupt autophagic balance, leading to cancer progression. Liu et al. summarized the molecular mechanisms linking lncRNAs to autophagy and their implications for cancer progression and treatment.

All these researches have shown that lncRNAs can regulate immune processes, influence drug resistance, and modulate TME, contributing to cancer progression and treatment outcomes. This compilation of studies underscores the significant roles of lncRNAs in various cancers, highlighting their potential as biomarkers and therapeutic targets. Collectively, as research on lncRNAs deepens, novel opportunities for enhancing cancer prognosis and therapeutic strategies are emerging.
